# Potential value of guard-wire technology in the interventional treatment for ostial coronary lesions

**DOI:** 10.1186/s12872-020-01779-5

**Published:** 2020-11-19

**Authors:** Xiaoqiong Wang, Xuemei Zong, Bingqiang Li, Zhanying Han, Xinjie Duan, Ying Li, Jing Zhang, Yaohui Wang, Yaoli Wang

**Affiliations:** 1 Department of Cardiology, The First People’s Hospital of Pingdingshan City (The First Affiliated Hospital of Pingdingshan University), 117 Youyue Road, Weidong District, Pingdingshan, 467000 Henan Province China; 2 Department of Cardiology, The Second People’s Hospital of Pingdingshan City, Pingdingshan, 467000 Henan Province China; 3 The First Affiliated Hospital of Zhengzhou University, Zhengzhou, 450052 Henan Province China; 4 ICU of Army Special Characteristic Center (Daping Hospital) of PLA, Chongqing, 400000 China

**Keywords:** Ostial lesions, Guard-wire technology, Aortasinus-in guard-wire technology, Branch guard-wire technology

## Abstract

**Background:**

To explore potential value of guard-wire technology during percutaneous coronary intervention (PCI) in patients with ostial coronary lesions.

**Methods:**

Patients, who underwent PCI, were collected between October 2011 and March 2017. Of the 141 patients, 63 (44.7%) have ostial lesions, and 78 (55.3%) have distal bifurcation sites. They were divided into group A (n = 71) and group B (n = 70). Group A received PCI after guard-wire technology. Group B were given balloon dilation and stent after placing guide wire through target lesion vessel. X-ray exposure time, contrast agent dosage, total PCI duration, pressure incarceration times, cases of malignant arrhythmia and cases of failed PCI of all patients were analyzed, respectively.

**Results:**

The general clinical characteristics includes patients age, sex ratio, the proportion of complications, smoking ratio and left ventricular ejection fraction of both groups was not significantly different. X-ray exposure time, contrast agent dosage, PCI total time, stent positioning time, pressure infestation frequency, arrhythmia frequency and complication number of group B were higher than those of group A. There is no case of malignant arrhythmia and case of failed PCI in group A, while there were five malignant arrhythmia and four failed PCI in group B. Contrast agent dosage and cases of failed PCI increased in group B compared with group A.

**Conclusion:**

The guard wire technology is safer and more feasible to patients with ostial coronary lesions who underwent PCI.

## Background

Ostial coronary lesions (AOL) are defined as a stenosis > 50% within 3 mm of the orifice in right coronary artery (RCA) or left main coronary artery (LMCA). The majority of ostial coronary lesions are due to atherosclerotic coronary artery disease. Fibrocellular and sclerotic fragments were the major tissue components in coronary lesions. An ostial lesion is characterized by a rigid fibrotic texture with pronounced sclerosis associated with a very high tendency to recoil, and this may lead to a higher complication rate and a higher rate of restenosis in primary interventional result. Despite the introduction of various new technologies, vascular lumen of the right coronary artery are more challenging for percutaneous interventional treatment with a higher restenosis rate [1].

Optimal ostial coronary lesions stenting requires placement of the entire circumference of the proximal stent edge within the aorto-ostial landing zone (AOLZ), which defined as the area along the axis of the coronary artery located within 1 mm of the aorto-ostial plane. When at least a segment of the circumference of the proximal stent edge is located proximal or distal to the AOLZ, it was diagnosed as stent geographic miss. Accurate Ostial coronary lesions stent implantation is crucial. In order to increase the stability and coaxiality of the catheter, reduce the dislocation of the catheter, prevent the catheter’s incorrect stent implantation leading to coronary artery and stent injury, reduce the incidence of complications, we summed up the "guard" wire technology (Guard-wire technology, GWT). "Guard" guide wire technology refers to the treatment of ostial coronary artery lesions or protection of mild lesions in the treatment of ostial lesions, through the target lesion before and after placing the main wire, we placed a second guide wire in the open proximal aortic sinus (Aortasinus-in Guard wire technology, AGWT) and branch "guard" technology (Branch Guard-wire technology, BGWT), which can be used as a guardian of the "guard" in these two situation [2, 3]. The second guide wire can be "guard" as a "buddy wire" by the implantation of the ostial vessel support to ensure further stability in the guide catheter.

## Methods

### Ethics approval and consent to participate

This study is a retrospective study and data analysis was performed anonymously.Each subject gave written informed consent in accordance with the Declaration of Helsinki, and this study was approved by The First People’s Hospital of Pingdingshan City (The First Affiliated Hospital of Pingdingshan University) Ethic Committee. The medical files described in our study are managed by the First People's Hospital of Pingdingshan City, and we have obtained the right to use the files.

### Study population and isolates

A total of 141 PCI patients (≤ 75 years), were chosen from the First People's Hospital of Pingdingshan City between October 2011 and March 2017. They were divided into group A (n = 71) and group B (n = 70). Group A were given PCI after using guard-wire technology. Group B were given balloon dilation and stenting after placing guide wire through target lesion vessel. Of the 141 patients, 63 (44.7%) had ostial lesions; 78 (55.3%) had distal bifurcation sites. Among the 141 patients, 83 males and 58 females were aged from 41 to 75 (62.8 ± 6.1) years. Inclusion criteria: (1) In accordance with the 2016 ACC/AHA Guideline, all consecutive patients of 18 years or older, who were admitted with stable angina pectoris (sAP), non ST-segment elevation acute coronary syndrome (NSTE-ACS) or ST-segment elevation myocardial infarction (STEMI) and underwent PCI in our institution, were included in the analysis. Exclusion criteria: (1) the presence of antiplatelet drugs taboo or cannot have long-term oral antiplatelet drugs, (2) severe liver and kidney dysfunction, (3) other serious diseases.

### Dual antiplatelet therapy before and after PCI

All patients were given preoperative or immediate aspiration of aspirin, enteric-coated tablets (300 mg, Bayer Pharmaceuticals), clopidogrel tablets (300 mg, France Sanofi-Aventis) or clopidogrel (AstraZeneca, 180 mg) before PCI. Tegeluo Luo (100 mg / time, 1 time/day), clopidogrel tablets (100 mg/times, 1 times/day) or tegrelol (90 mg/time, 2 times/day) after PCI for at least 1 year. Angiographic follow-up was scheduled according to local guidelines; or noninvasive evaluation; or clinical presentation suggested ischemia one month after surgery.

### "Guard" guide wire technology

**Fig. 1:**
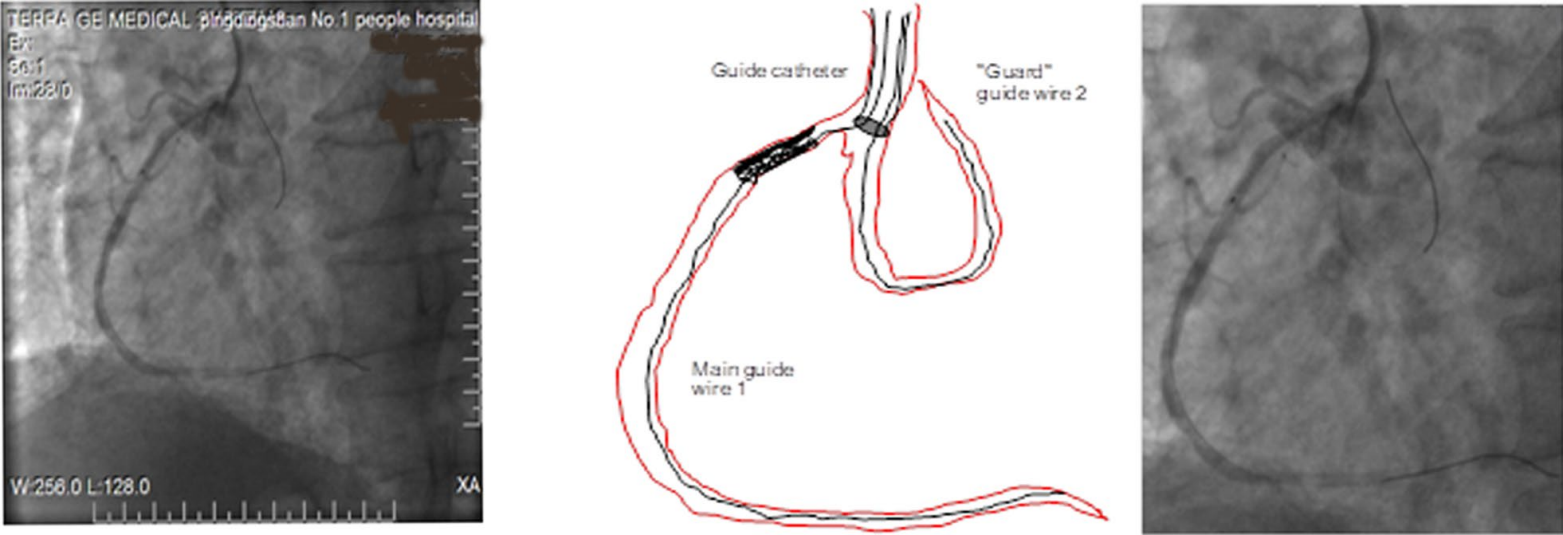
Sinus guard-wire technology. Ostial lesion of the right coronary artery, the main guide wire was passed through the lesion to the distal, the second guard wire was placed in the right aortic sinus. The Sinus guard-wire technique for an ostial bifurcation lesion of the right coronary artery. (1): the main guide wire was passed through the lesion to the distal, and the second guard wire was placed within the right aortic sinus. (2): the stent is placed into the guide catheter over both the main wire and the guard wire which resides in the other branch. (3): The stent is placed into the ostial lesion. (4): The stent advancement is stopped by the guard wire, and then, deployed at low atmospheres. The guard wire is then removed, and the stent is deployed at high atmospheres

**Fig. 2:**
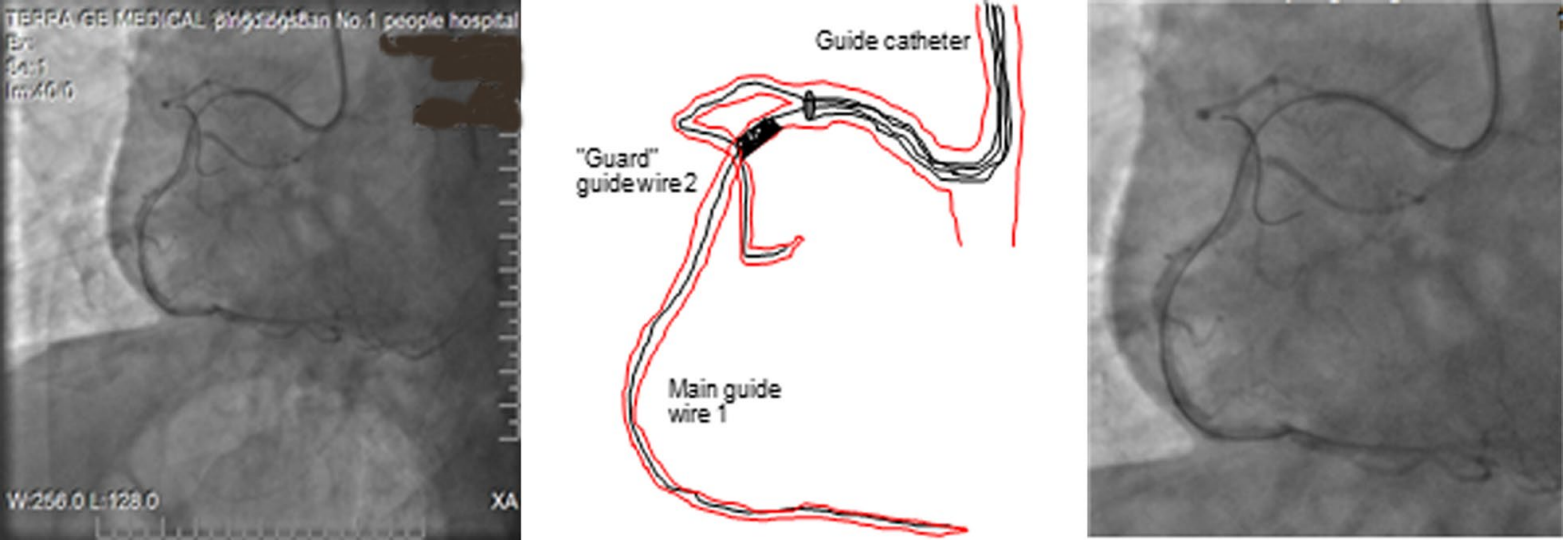
Branch guard-wire technology. Ostial lesion of the right coronary artery, the main guide wire was passed through the lesion to the distal, and the second guard wire was placed in the right coronary artery proximal branch. The branch guard-wire technology for an ostial bifurcation lesion of the right coronary artery. (1): the main guide wire was passed through the lesion to the distal, the second guard wire was placed in the right aortic artery proximal branch. (2): The stent is placed into the guide catheter over both the main wire and the guard wire which resides in the proximal branch. (3): The stent is placed into the ostial lesion. (4): The stent advancement is stopped by the guard wire, and then, deployed at low atmospheres. The guard wire is then removed, and the stent is deployed at high atmospheres

"Guard" guide wire technology (GWT) refers to place protective guide wire in the ostial lesion proximal sinus or branch in the treatment of coronary artery disease, which is divided into AGWT and BGWT. AGWT can increase the passive support force, prevent the catheter implanted injuring coronary artery, auxiliary stent positioning, et al. BGWT can play as an anchor catheter to prevent catheter dislocation, improve catheter coaxial reduction catheter incarcerated, auxiliary stent positioning, thereby prevent catheter implanted deep insertion of the stent into the vessel beyond the aorto-ostial plane. The specific clinical method is as follows: ostial lesion of the right coronary artery, the main guide wire was passed through the lesion to the distal, the second guide wire ("guard" guide wire) was placed in the right aortic sinus (see Fig. 1) or right coronary artery proximal branch (see Fig. 2). Similarly, the left main lesion, the second guide wire was placed in the left aortic sinus. Before the descending branch lesions, the second guide wire was placed in the circumcision or anterior descending branch of the lesion proximal branch. Roundabout branch of the lesion, the second guide wire was placed in the anterior descending artery or circumflex artery proximal branch.

### Variables

Clinical presentation, demographics, laboratory results and CAG characteristics, treatments were collected through medical records and angiographic reviews. The detail of angiographic features included lesion location, lesion length, percentage of lesion vessel stenosis, percentage of PCI, intimal dissection or intramural hematoma and CAG imaging types.

All PCI patients’ X-ray exposure time, contrast agent dosage, total PCI duration, pressure incarceration times, cases of malignant arrhythmia and cases of failed PCI were analyzed, respectively. Stent positioning time is the time when the stent began to guide from the catheter to the release of the balloon. PCI total time is the time when the stent began to guide from catheter into the arterial sinus, to the end of the last angiography. Malignant arrhythmia refers to the sinus arrest, the atrioventricular block is higher than degree II, ventricular tachycardia, ventricular fibrillation. Pressure incarceration is defined as the guideline head pressure is declined 50% than the aortic pressure or pressure waveform ventricularization. Perioperative operation-related complications refer to the absence of stent coverage at the lesion, incomplete scaffold coverage, vascular tearing, acute thrombosis within 6 h after stenting. The case of combining other guide wire technologies refers to the application cases of combining dual guide wire technology, edge protection guide wire technology and other guide wire technologies during the intervention process.

### Statistical analysis

Data were presented as mean ± standard deviation (SD) for continuous variables and as frequency (%) for categorical variables. Student's t-test was used for comparison of normally distributed continuous data. The count data is expressed by the number of cases (constituent ratio). The χ^2^ test was used to compare difference between groups. Statistical analysis was performed with the SPSS version 18.0 (SPSS Inc., Chicago, IL, USA). *P* < 0.05 was considered statistically significant.

## Results

### The baseline clinical characteristics of PCI patients using the protected line technology and using the standard coronary stent technology

The general clinical characteristics includes patients age, sex ratio, the proportion of complications, smoking ratio and left ventricular ejection fraction of the two groups have no statistically significant difference (*P* > 0.05) (Table 1).

**Table 1:** Baseline clinical characteristics of PCI patients in the two groups

Characteristics	A group (*n* = 71)	B group (*n* = 70)	*P* value
Age (years)	62.9 ± 5.9	63.1 ± 6.9	0.31
Male (n, %)	43 (60.6)	40 (57.1)	0.68
Hypertension (n, %)	38 (53.5)	32 (45.7)	0.35
Diabetes (n, %)	21 (29.6)	18 (25.7)	0.61
Hyperlipidemia	39 (54.9)	33 (47.1)	0.36
Smoking	37 (52.1)	34 (48.6)	0.67
LVEF	57.5 ± 5.0	56.3 ± 5.1	0.77

*LVEF* left ventricular ejection fraction

### The comparison of different characteristics of lesions in two groups

There was no significant difference between the characteristics of lesions in two groups (including surgical-related indicators, target lesion maximum stenosis, target lesion length, target vessel diameter, cases combined with the application of other guide wire technology, ostial lesions and distant lesions) (*P* > 0.05). X-ray exposure time, contrast agent dosage, PCI total time, stent positioning time, pressure infestation frequency, arrhythmia frequency and complication number of group B were higher than those of group A (*P* < 0.05)) (Table 2). In group A, there was 1 patient with malignant arrhythmia; 2 patients with stent were not completely covered lesions which need to add another stents. In group B, there were 8 patients with malignant arrhythmia; 5 patients with stent were not completely covered lesions which need to add another stents; 2 patients with coronary endometrial tear, and 2 patients with stent acute thrombosis.

**Table 2:** lesions characteristics, surgical-related indicators between Group A and B

Variables	A group (*n* = 71)	B group (*n* = 70)	*P* value
Maximum stenosis (%)	83.6 ± 5.1	83.0 ± 5.4	0.41
Target lesion length (mm)	24.0 ± 4.1	24.7 ± 3.9	0.65
Target vessel diameter (mm)	3.3 ± 0.32	3.2 ± 0.38	0.14
Number of patients with ostial lesions (n, %)	32 (45.1)	31 (44.3)	0.93
Number of patients with distant lesions (n, %)	39 (54.9)	39 (55.7)	0.93
Number of cases combined with other guide wire technology (n, %)	9 (12.7)	11 (15.7)	0.61
X-ray exposure time (min)	6.0 ± 0.84	9.0 ± 1.2	0.005
The contrast agent (ml)	85.8 ± 6.2	97.1 ± 10.1	0.000
PCI total time (min)	11.2 ± 1.1	16.9 ± 1.8	0.000
Stent positioning time (min)	1.1 ± 0.2	2.5 ± 0.1	0.02
Number of pressure marshals (n, %)	4 (5.6)	13 (18.6)	0.018
Arrhythmia (n, %)	1 (1.4)	8 (11.4)	0.015
Complications (n, %)	2 (2.8)	9 (12.9)	0.026

## Discussion

Percutaneous coronary intervention of an ostial lesion is a challenging procedure, even for experienced operators, especially if the angle between the bifurcating vessels is less than 75°, or if the lesion is 0.0.1 (according to Medina). Inaccurate stent positioning may be a major cause for suboptimal outcomes of aorto-ostial interventions [4]. Historically, treatment of ostial lesion was associated with lower procedural success and higher adverse outcomes compared to non-ostial lesions [5]. This was attributed to guide catheter trauma to the ostium, inadequate guide catheter support, increased elastic recoil and a propensity for intimal disruption and coronary dissection. Stable guide catheter operation and accurate stent positioning are the main difficulty in interventional treatment of coronary artery lesions. If surgeons cannot accurately locate the stent and all cover the disease, it may lead to release of stents and other issues. Guide catheter stability and coaxial poverty may be associated with extension of intervention time that affecting the quality of surgery, or even leading to surgical failure, causing non-target lesions vascular injury. In order to ensure the effective positioning and expansion of the stent, it is a common choice to guide the catheter from the coronary artery ostial, especially in the treatment of left main or right coronary artery lesions. However, it may affect surgeons’ judgments of the coronary artery image, increas catheter instability and accurate placement. A number of studies have shown that Szabo technique uses two-wire anchors to pinpoint the ostial lesion stent, thereby preventing deep insertion of the stent into the vessel beyond the ostial plane [6–9]. Despite reducing the incidence of adverse events and increasing the success rate, it has been found that the technology may lead to stent deformation and dislodgement [10–12]. In addition, asymmetric damage of the stent and difficult of accurate stent positioning may increasing the risk of stent shedding. On top of that, it bothers surgeons when there is need to open mild lesions of coronary artery involved in the treatment of ostial distal lesions. The problem that the guide wire and balloon through the ostial lesions, the relative movement of the balloon and the catheter caused coronary artery lesion injury may bring trouble to surgeons. During the clinical intervention process, we found that "guard" guide wire technology can be more effective to improve the stability of the catheter and coaxial, reduce swing of the guide wire, balloon and other in the blood vessels, which facilitates the accurate positioning of the stent and reduces the inevitable ostial damage.

In this study, we found that X-ray exposure time, the amount of contrast agent application, the number of pressure instigation, stent positioning time and PCI total time, the number of intraoperative malignant arrhythmias and perioperative management-related complications of group B were higher than those of group A (all *P* < 0.05). This study suggests that the support and coaxiality of the guide catheter was improved by use of the "Guard" guide wire. During the interventional treatment of the ostial lesion, the "Guard" guide wire technique makes the catheter more stable and easier to operate; reduced the X-ray exposure time, contrast agent application, stent positioning time and PCI total time; makes surgical operation process smoother, and improve the catheterization. In addition, improvement of catheter support and coaxiality boosts the accurate stent positioning. The stable operation of the guide catheter, the smooth operation, and satisfying placement all contribute to the decrease the number of intraoperative malignant arrhythmia and perioperative related complications. In the approach of mild distant lesions treatment, the improvement of "guard" guide wire guide catheter support and coaxial improvement reduce "swing" amplitude of the catheter in coronary heart disease caused by cardiac contraction, coronary blood flow, making it easier for the balloon and guide wire through the lesion; reducing the collision or friction of the endometrium of the coronary lesion; reducing the incidence of complications was reduced. During the course of operation, "Guard" guide wire technology can have a more flexible application. "Guard" guide wire technology can be combined or alternately. Between the "Guard" guide wire technology and double guide wire technology, edge support wire technology, and other guide wire technology can also be used in conjunction or alternately. This flexible application can guarantee PCI patients greater benefits.

The challenges of PCI of an ostial lesion included not only upon the lesion location but also on the plaque burden and lesion morphology. Ostial lesions are less compliant as compared with non-ostial lesions due to increased fibrosis, calcification, and muscular/elastic tissue. Complete stent coverage of ostial lesions can be problematic, and inaccurate stent placement leading to higher rates of restenosis. This study shows that in the treatment of coronary artery lesions and mild coronary artery distal lesions, the guard "guide wire technology can play as an anchor catheter to prevent catheter dislocation, improve catheter coaxial, reduce catheter incarcerated, conduct auxiliary stent positioning, prevent deep insertion of stent from damaging coronary artery, reduce the operation time, improve the success rate of surgery, reduce the risk of surgery and reduce the incidence of complications. It can be combined with other wire techniques or alternating application.

## Conclusions

Above all, it has a good clinical application value and "guard" guide wire technique in clinical practice is of good safety and feasibility.

## Data Availability

The datasets used and/or analyzed during the present study are available from the corresponding author on reasonable request.

## Abbreviations

PCI: Percutaneous coronary intervention
AOL: Ostial coronary lesions
RCA: Right coronary artery
LMCA: Left main coronary artery
AOLZ: Aorto-ostial landing zone
AGWT: Aortasinus-in Guard wire technology
BGWT: Branch Guard-wire technology
NSTE-ACS: Non ST-segment elevation acute coronary syndrome
STEMI: ST-segment elevation myocardial infarction
GWT: "Guard" guide wire technology
SD: Standard deviation
